# Fuelled or Fooled? Examining the Evidence and Mechanisms Behind Ultra-High Carbohydrate Intake in Endurance Athletes

**DOI:** 10.1007/s40279-026-02462-z

**Published:** 2026-06-08

**Authors:** Daniel J. Plews, Paul D. Booth, Taj Krieger, Ed Maunder

**Affiliations:** 1 Sports Performance Research Institute New Zealand, Auckland University of Technology, Auckland, New Zealand; 2 Research Institute for Sport, Physical Activity, and Leisure, Carnegie School of Sport, Leeds Beckett University, Leeds, United Kingdom of Great Britain and Northern Ireland; 3 California State Polytechnic University, Humboldt, Arcata, CA USA; 4 Pillar Performance, Head of Research, Gold Coast, QLD Australia

## Abstract

Ultra-high carbohydrate (U-HC) ingestion strategies, typically > 90 g·h⁻^1^, have gained remarkable traction among elite endurance athletes in recent years. This rise has been fuelled by anecdotal reports linking aggressive carbohydrate fuelling with superior race execution, and record-breaking performances. However, the scientific literature supporting these practices remains limited, and current frameworks used to explain carbohydrate-mediated performance effects, such as muscle glycogen sparing, maintenance of blood glucose or maximisation of exogenous carbohydrate oxidation, do not fully account for the reported U-HC benefits. Indeed, available studies reveal a disconnect between metabolic data and the real-world adoption of intakes far beyond established oxidation ceilings. We examine the existing research on carbohydrate ingestion during endurance exercise, highlighting the metabolic responses and performance outcomes. We identify how traditional mechanisms have been interpreted to justify escalating carbohydrate intake and discuss the constraints of current methodologies in detecting meaningful performance benefits at ultra-high ingestion rates. The article then introduces emerging, yet largely untested, possible mechanistic models that may underpin the perceived utility of U-HC fuelling, including shifts in substrate selection, enhanced lactate shuttling and potential central effects associated with carbohydrate availability. Finally, we explore the relevance of inter-individual variation in carbohydrate oxidation capacity and the implications for developing practical guidelines for athletes. By integrating physiological, metabolic and perceptual perspectives, this current opinion outlines why U-HC strategies have gained prominence and identifies key areas where further research is required to clarify their role within contemporary endurance performance for the masses.

**Supplementary Information:**

The online version contains supplementary material available at https://doi.org/10.1007/s40279-026-02462-z.

## Key Points

**Table:** 

Ultra-high carbohydrate ingestion (> 90 g·h⁻^1^) during endurance exercise remains poorly evidenced, with supporting data largely derived from anecdotal reports and elite athlete practice rather than controlled research.
Despite well-established relationships between carbohydrate ingestion and exogenous oxidation rates, data do not consistently support dose-dependent performance benefits beyond 90 g·h⁻^1^, nor does increasing exogenous carbohydrate intake appear to spare endogenous carbohydrate utilisation; three alternative mechanistic explanations for potential performance enhancement are proposed.
Blanket promotion of ultra-high carbohydrate fuelling across all athlete populations is not currently justified; while some elite athletes may derive individual benefit, a cautious and individualised approach is strongly recommended.

## Introduction

There has been growing interest in ultra-high carbohydrate (U-HC) intake during endurance exercise, with studies exploring ingestion rates of up to 120 g·h⁻^1^ [1, 2]. Anecdotal reports suggest that elite endurance athletes, in sports such as marathon, ultramarathons, professional cycling and long-course triathlons, are adopting aggressive carbohydrate fuelling strategies during competition. These practices have been associated with record-breaking performances and improved race-day execution, leading to what has been widely described as a ‘carbohydrate revolution’ in endurance sport [40]. However, despite this surge in popularity, the scientific evidence supporting the physiological advantages of U-HC ingestion remains limited.

Researchers have attempted to explain the effects of U-HC ingestion rates during endurance exercise on performance through the lens of energy availability [3], muscle glycogen sparing [4], prevention of hypoglycaemia [5] and/or enhanced exogenous carbohydrate oxidation [6]. However, the use of U-HC intake during endurance exercise for these reasons may not stand up to scrutiny. For example, a recent meta-analysis suggests that the muscle glycogen-sparing effect of carbohydrate ingestion during exercise is small and not dose-dependent, at least within the range of ingestion rates studied [7]; blood glucose concentrations during endurance exercise can be stabilised at more moderate carbohydrate ingestion rates [8]; and the extra exogenous carbohydrate oxidation at ingestion rates above 90 g·h^−1^ seems only to blunt fat oxidation rates. Carbohydrate ingestion during endurance exercise suppresses hepatic glucose output [9] and therefore attenuates exercise-induced liver glycogen depletion [10], at moderate ingestion rates. Indeed, it has been recently suggested that the performance benefit of carbohydrate ingestion during prolonged exercise appears to be primarily mediated by the maintenance of euglycemia and the prevention of exercise-induced hypoglycaemia, rather than by dose-dependent increases in carbohydrate oxidation [11]. Together, these findings suggest that escalating carbohydrate intake to U-HC levels is unlikely to confer additional hepatic, muscle or systemic metabolic benefits beyond what is achieved at more moderate ingestion rates. Nevertheless, interest remains in the potential for U-HC intake during endurance exercise to improve performance.

U-HC intakes have been reported within elite sport since at least 2009, when Pfeiffer et al. [12] reported intakes of up to 120 g·h⁻^1^, associating high carbohydrate intakes with faster finishing times during international Ironman events. Viribay et al. [13] and colleagues reported a significant positive effect on recovery and reductions in exercise-induced muscle damage in elite trail runners ingesting carbohydrates at 120 g·h⁻^1^ compared with 90 and 60 g·h⁻^1^. At the same time, a significant body of evidence supporting the ingestion of multiple transportable carbohydrates at higher than previously recommended ingestion rates (~ 90 versus 60 g·h⁻^1^) to increase exogenous oxidation rates accumulated [14]. These observations likely laid the foundations for the current U-HC revolution. However, these early studies did not support specific performance benefits of U-HC ingestion rates. Pfeiffer et al. [12] also observed equivalent best performance times of athletes ingesting 80 g·h⁻^1^, and importantly, both Pfeiffer et al. [12] and Viribay et al. [13] did not find any significant improvement in performance times, only markers of recovery. Therefore, there appears to be a disconnect between what is reported in the field and what is observed in the laboratory, both metabolically and in terms of performance outcomes with U-HC intakes.

The purpose of this current opinion is threefold. First, we review the literature on carbohydrate ingestion during exercise, with a focus on metabolic responses and performance. Second, we explore potential mechanisms, beyond the prevention of hypoglycaemia and preservation of endogenous stores, that may explain the anecdotal benefits of U-HC ingestion rates. Finally, we consider whether there is sufficient justification to recommend U-HC ingestion rates in general guidelines to athletes. We define U-HC ingestion as intake rates exceeding 90 g·h⁻^1^, which surpasses the upper limit currently recommended in established sports nutrition guideline [15].

### Ultra-High Carbohydrate Ingestion Does Not Further Spare Endogenous Glycogen

Research into the optimal rate of carbohydrate ingestion during endurance exercise has primarily focused on identifying the ingestion rate that maximises exogenous carbohydrate oxidation [16, 17]. The rationale is that exogenous carbohydrate oxidation reduces endogenous carbohydrate oxidation and preserves finite liver and skeletal muscle glycogen stores, the depletion of which are key mechanisms of fatigue during prolonged exercise [18–20]. Early studies demonstrated that combining glucose with fructose, absorbed via separate intestinal transporters (SGLT1 and GLUT5 respectively), increases exogenous oxidation rates up to ~ 90 g·h⁻^1^ [10, 21, 22]. Ingestion of multiple transportable carbohydrates therefore underpins most contemporary fuelling guidelines [14].

A critical question in making the case for U-HC ingestion rates is whether increasing carbohydrate ingestion beyond 90 g·h⁻^1^ translates into further reductions in endogenous carbohydrate oxidation. The available evidence suggests it does not. Podlogar et al. [6] provided the clearest demonstration of this: Carbohydrate ingestion at 120 g·h⁻^1^ increased exogenous carbohydrate oxidation rates compared with 90 g·h⁻^1^, but there was no corresponding reduction in endogenous carbohydrate oxidation. Ravikanti et al. [2] corroborated this finding, as endogenous carbohydrate oxidation rates were not different between 60, 90, and 120 g·h⁻^1^ ingestion rates, even in elite marathon runners achieving some of the highest exogenous oxidation rates ever recorded in the 120 g·h⁻^1^ trial. King et al. [23] went further, showing that 112.5 g·h⁻^1^ actually led to greater muscle glycogen oxidation compared with lower doses.

Moreover, even if exogenous carbohydrate oxidation rates rise when increasing carbohydrate ingestion beyond 90 g·h⁻^1^, oxidation efficiency, or the proportion of ingested carbohydrates that are oxidised, is reduced. Podlogar et al. [6] observed that oxidation efficiency dropped from ~ 86% at 90 g·h⁻^1^ to ~ 76% at 120 g·h⁻^1^, leaving ~ 30 g·h⁻^1^ unoxidised. Importantly, if exogenous oxidation is quantified using cumulative oxidation over time rather than extrapolated from peak instantaneous rates, the proportion of ingested carbohydrate that remains unoxidised is even greater [24, 25]. Therefore, assuming oxidation rates match ingestion rates throughout exercise may substantially overestimate the contribution of exogenous carbohydrate to energy production at ultra-high intake rates.

If not all ingested carbohydrate is oxidised, what is its fate? Rehrer et al. [26] observed a significant correlation between exogenous carbohydrate oxidation and carbohydrate emptied from the stomach, suggesting that a large proportion may remain unabsorbed in the gastrointestinal tract. This means that gastrointestinal issues may be an important concern when consuming carbohydrate at ultra-high rates. Higher carbohydrate concentrations (> 8–10%) slow gastric emptying and increase symptoms of fullness, bloating, and nausea [27]. While U-HC intakes have been observed to be well-tolerated in some studies [6, 25, 28], the shorter trial durations used in laboratory settings may be inadequate to capture the timepoint at which gastrointestinal distress typically emerges during ultra-endurance events [12, 29], meaning tolerance is likely considerably overstated in current literature.

Interestingly, Ahlborg and Felig [30] suggest the liver is a major site of glucose retention, with excess carbohydrates retained in the splanchnic bed and gastrointestinal tract. This retained carbohydrate may be directed towards more rapid glycogen synthesis following exercise, which could partly explain the improved recovery markers observed by Viribay et al. [13] at 120 g·h⁻^1^ compared with 90 and 60 g·h⁻^1^. Alternatively, additional carbohydrate intake around exercise may improve within-day energy balance, reducing endocrine markers associated with impaired recovery [31].

The glucose-to-fructose ratio may modulate these effects. In contrast to King et al. [23], Podlogar et al. [6] did not observe increased endogenous carbohydrate oxidation at 120 versus 90 g·h⁻^1^, perhaps owing to their use of a more balanced ratio (1:0.8), suggesting that a higher proportion of fructose may reduce SGLT1 saturation and improve oxidation efficiency. Ravikanti et al. [2] further support this, reporting average whole-body carbohydrate oxidation rates of 1.68 ± 0.16 g·min⁻^1^ at 120 g·h⁻^1^ using a 1:1 maltodextrin-to-fructose ratio. However, even under these optimised conditions, endogenous carbohydrate oxidation was not different across doses.

Therefore, the data suggest that any extra exogenous carbohydrate oxidation achieved with U-HC ingestion rates proportionally reduces fat oxidation and therefore fails to further reduce endogenous carbohydrate oxidation. Accordingly, if the rationale for increasing carbohydrate oxidation rates beyond 90 g·h⁻^1^ is to reduce endogenous carbohydrate oxidation, the available evidence does not support it.

### There Is a Lack of Evidence for Improved Performance with Ultra-High Carbohydrate Ingestion Rates

Sports nutrition research has mostly emphasised physiological parameters, such as substrate oxidation or metabolic responses, rather than direct measures of performance. Accordingly, despite our ability to predict the oxidation response to various carbohydrate doses with reasonable certainty, our capacity to predict the performance impact of those doses remains limited, owing to limited research.

The studies that have been conducted do not support a dose–response relationship between carbohydrate ingestion rates and performance. Smith et al. [32] had 51 cyclists complete four trials consisting of 2 h of cycling followed by a 20-km time trial (TT). Participants were assigned to receive no carbohydrate or one of three ingestion rates drawn from a range between 10 and 120 g·h⁻^1^. A mixed-model analysis predicted performance benefits up to ~ 78 g·h⁻^1^, beyond which diminishing returns and even performance decrements were projected. However, the relationship between ingestion rate and TT performance was flat between ~ 30 and 90 g·h⁻^1^. Supporting a dose–response relationship within conventional intake ranges, Fell et al. [33], demonstrated progressively greater exercise capacity with increasing carbohydrate ingestion across 0, 45 and 90 g·h⁻^1^. Notably, however, ingestion rates above 90 g·h⁻^1^ were not examined in that study, and whether this dose–response relationship extends into U-HC ingestion rates remains to be established. Similarly, King et al. [23] had trained cyclists complete 2 h of cycling at 77% of V̇O_2_max followed by a 30-min TT, under conditions of 0, 60, 75, 90 and 112.5 g·h⁻^1^ of carbohydrate ingestion. Although performance was enhanced by carbohydrate ingestion relative to placebo, no statistically significant differences were observed between the carbohydrate doses. These findings were supported in a follow-up study [34]. This mismatch between ingestion rate, exogenous carbohydrate oxidation and performance outcome is notable. Higher intake does not appear to translate into proportionally greater oxidation or measurable performance benefit. Taken together, while carbohydrate ingestion consistently improves performance relative to placebo, clear evidence for a dose–response effect beyond relatively modest intakes (~ 30–90 g·h⁻^1^) is lacking. Across multiple performance studies, increasing carbohydrate ingestion above these levels does not consistently produce additional performance benefits, despite higher rates of exogenous carbohydrate oxidation [11].

### Ultra-High Carbohydrate Ingestion Rates and Gut Training

Historically, the rationale for increasing carbohydrate intake during endurance training has centred on improving gastrointestinal tolerance (‘gut-training’). This involves the repeated ingestion of high carbohydrate loads during exercise, with the aim of enhancing gastric emptying, intestinal absorption and ultimately reducing gastrointestinal distress during competition. While some evidence suggests that this approach may improve subjective tolerance and reduce markers of carbohydrate malabsorption, particularly where fructose-specific adaptations in gastric emptying rate have been observed [35, 36], there is limited support for enhancement in exogenous carbohydrate oxidation capacity [37, 38]. One exception is Cox et al. [39], who demonstrated that daily training with high carbohydrate availability increased exogenous carbohydrate oxidation, most likely through upregulation of intestinal SGLT1 transporters; however, this finding has not been consistently replicated.

## Mechanisms for Why Ultra-High Carbohydrate Ingestion Rates during Endurance Exercise may be Beneficial

This raises a key question: What is driving the trend towards ever-increasing carbohydrate intake, and could this truly underpin the surge in world-class performances observed in recent years [40]. We propose three possible mechanisms through which U-HC ingestion could improve performance.

### Improved Oxidative Efficiency Through Substrate Shift?

Carbohydrate oxidation yields greater energy per litre of oxygen consumed than fat oxidation. Oxidising 1 L of oxygen with carbohydrates yields approximately 5.05 kcal, whereas fat oxidation yields closer to 4.69 kcal per litre of oxygen [41]. This has implications during exercise intensities when oxygen availability is a factor, and events that take place over extended (ultra) duration. An athlete who relies more heavily on carbohydrates can achieve the same mechanical output with less oxygen cost, improving performance capacity and possibly delaying fatigue. Lukasiewicz et al. [42] recently tested this hypothesis, modelling carbohydrate ingestion rates to perform a sub-2 marathon. They suggested an increased need for carbohydrate ingestion > 90 g·h⁻^1^ for all runners, and up to 130 g·h⁻^1^ for some female runners.

This principle has been consistently observed across multiple shorter-term ketogenic low-carbohydrate, high-fat (LCHF) diets in athletes. Despite increased rates of fat oxidation, athletes exhibit higher oxygen consumption (VO_2_) at the same absolute workloads when adapted to LCHF diets [43–45]. This elevated metabolic cost of fat oxidation may offset the potential benefits of greater fat availability and is a direct result of increased fat oxidation. However, while some studies have shown this to impair endurance performance under race-like conditions [45, 46], systematic reviews have found unchanged performance [47].

It is established that increasing exogenous carbohydrate intake during exercise is generally associated with a suppression of fat oxidation [7]. Recently, Noakes et al. [11] provided direct support for this, demonstrating that increasing carbohydrate ingestion produces a linear suppression of fat oxidation across a broad range of intake rates. Consistent with this, the data presented in Table 1 show that reductions in fat oxidation relative to placebo increase in parallel with escalating carbohydrate ingestion. A quantitative analysis of these data reveals a large negative correlation between carbohydrate intake and fat oxidation (*r* =  − 0.58; 90% confidence interval [CI]: − 0.73 to − 0.37), indicating that greater carbohydrate ingestion progressively shifts substrate utilization from fat towards carbohydrates.

**Table 1:** Exogenous carbohydrate ingestion rates and changes in fat and carbohydrate oxidation

Study	CHO ingestion rate (g·min^−1^)	CHO ingestion rate (g·h^−1^)	CHO solution	Peak Exo CHO Ox (g·min^−1^)	Avg Exo CHO Ox (g·min^−1^)	Avg Endo CHO Ox (g·min^−1^)	Fat Ox rate (g·min^−1^)	Ox efficiency (%)	Δ Fat Ox (g·min^−1^)	Δ Fat Ox (%)	↑ Total CHO Ox (g·min^−1^)	↑ Total CHO Ox (%)	Δ Endo CHO Ox (g·min^−1^)	Δ Endo CHO Ox (%)
	Amount	Amount	(Ratio)	Mean (± SD)	Mean (± SD)	Mean (± SD)	Mean (± SD)	(Mean)	(Mean)	(Mean)	(Mean)	(Mean)	(Mean)	(Mean)
Trommelen et al. (2017) [75]	1.8	108	GLU:FRUC 2:1	1.4 ± 0.06	1.19 ± 0.12	0.73 ± 0.12	0.79 ± 0.05	66.1	− 0.26	− 32.48	0.76	65.73	0.43	− 36.85
Trommelen et al. (2017) [75]	1.8	108	GLU	0.96 ± 0.06	0.82 ± 0.16	0.78 ± 0.12	0.88 ± 0.07	45.7	− 0.17	− 18.86	0.44	37.72	0.39	− 33.19
Trommelen et al. (2017) [75]	1.8	108	SUC:GLU 1.14:0.6	1.29 ± 0.07	1.13 ± 0.21	0.84 ± 0.14	0.74 ± 0.08	62.8	− 0.31	− 41.5	0.81	69.4	0.33	− 28.02
Trommelen et al. (2017) [75]	0	0	Water			1.16 ± 0.29	1.04 ± 0.09							
Mears et al. (2020) [74]	1	60	DEX:GLU 1:1.2	0.68 ± 0.14	0.38 ± 0.11	1.78 ± 0.45	0.7 ± 0.18	38						
Mears et al. (2020) [74]	1	60	DEX:GLU 1:1.2	0.61 ± 0.14	0.31 ± 0.11	1.92 ± 0.4	0.65 ± 0.15	31						
O’Brien & Rowlands (2011) [86]	1.8	108	MALTO:FRUC 1:2		1.04	0.95	0.59	57.8	− 0.39	− 66.1	1.13	131.4	− 0.09	10.47
O’Brien & Rowlands (2011) [86]	1.8	108	MALTO:FRUC 0.8:1		1.14	0.89	0.61	63.3	− 0.37	− 60.66	1.17	136.05	− 0.03	3.49
O’Brien & Rowlands (2011) [86]	1.8	108	MALTO:FRUC 1.25:1		1.05	0.82	0.62	58.3	− 0.36	− 58.06	1.01	117.44	0.04	− 4.65
O’Brien & Rowlands (2011) [86]	0	0	Water			0.86	0.98							
Röcker et al. (1996) [73]	0.3	18	GLU	0.36 ± 0.05	0.21 ± 0.05									
Narang et al. (2021) [85]	1.2	72	DEX	0.83 ± 0.15	0.5 ± 0.13			41.7						
O’Brien, et al. (2013) [72]	1.5	90	FRUC:MALTO–GLU 0.5:1		0.94	1.01	0.71	62	− 0.14	− 19.72	0.31	18.9	0.63	− 38.41
O’Brien, et al. (2013) [72]	1.5	90	FRUC:MALTO–GLU 0.8:1		1.1	1.04	0.64	74	− 0.21	− 32.81	0.5	30.49	0.6	− 36.59
O’Brien, et al. (2013) [72]	1.5	90	FRUC:MALTO 1.25:1		1.03	1.39	0.64	69	− 0.21	− 32.81	0.78	47.56	0.25	− 15.24
O’Brien, et al. (2013) [72]	0	0	Water			1.64	0.85							
Adopo, et al. (1994) [81]	0.42	25	GLU		0.32 ± 0.02			75.6						
Adopo, et al. (1994) [81]	0.42	25	FRUC		0.32 ± 0.01			76.7						
Adopo, et al. (1994) [81]	0.83	50	GLU		0.49 ± 0.07			58.3						
Adopo, et al. (1994) [81]	0.83	50	FRUC		0.46 ± 0.02			55.2						
Adopo, et al. (1994) [81]	0.83	50	GLU:FRUC 1:1		0.74 ± 0.06			88.7						
Pfeiffer, et al. (2011) [71]	1.5	90	MALTO:FRUC 2:1	1.25 ± 0.1	1.14 ± 0.1	0.67 ± 0.24	0.71 ± 0.15	76	− 0.25	− 35.21	0.58	47.15	0.56	− 45.53
Pfeiffer, et al. (2011) [71]	1.5	90	MALTO:FRUC 2:1	1.19 ± 0.08	1.08 ± 0.11	0.86 ± 0.32	0.61 ± 0.19	72	− 0.23	− 37.7	0.58	42.65	0.5	− 36.76
Pfeiffer, et al. (2011) [71]	0	0	Water			1.23 ± 0.47	0.96 ± 0.24							
Pfeiffer, et al. (2011) [71]	0	0	Water			1.36 ± 0.4	0.84 ± 0.23							
Hulston, et al. (2009) [82]	0.8	48	GLU	0.6 ± 0.07	0.58 ± 0.05	1.48 ± 0.14	0.58 ± 0.07	72.5	− 0.15	− 25.86	0.35	20.47	0.23	− 13.45
Hulston, et al. (2009) [82]	0.8	48	GLU:FRUC 2:1	0.57 ± 0.06	0.56 ± 0.06	1.58 ± 0.17	0.58 ± 0.07	70	− 0.15	− 25.86	0.43	25.15	0.13	− 7.6
Hulston, et al. (2009) [82]	0	0	Water			1.71 ± 0.05	0.73 ± 0.04							
Pettersson, et al. (2020) [70]	1.58	94.8	MALTO:FRUC 1:0.7	1.34 ± 0.16	0.95 ± 0.08	.93 ± 0.29	0.82 ± 0.14	60.1						
Pettersson, et al. (2020) [70]	1.58	94.8	MALTO:SUC 1:0.25	1.23 ± 0.12	0.9 ± 0.08	1.0 ± 0.41	0.79 ± 0.17	57						
Sutehall, et al. (2022) [90]	1.17	70.2	MALTO:FRUC 1:0.7	0.63 ± 0.07										
Hawley, et al. (1992) [69]	2	120	GLU	0.9 ± 0.09	0.55 ± 0.1			27.4						
Hawley, et al. (1992) [69]	2	120	MALTOSE	1.0 ± 0.09	0.57 ± 0.09			28.3						
Hawley, et al. (1994) [68]	2	120	GLU	0.98 ± 0.12	0.49 ± 0.06		0.23 ± 0.08	24.5						
Jentjens, et al. (2005a) [67]	2.4	144	GLU:FRUC 1:1	1.75 ± 0.11	1.49 ± 0.08	1.07 ± 0.15	0.51 ± 0.04	62.1	− 0.46	− 90.2	1.01	65.16	0.48	− 30.97
Jentjens, et al. (2005a) [67]	2.4	144	GLU	1.06 ± 0.04	0.99 ± 0.06	1.26 ± 0.12	0.67 ± 0.05	41.3	− 0.3	− 44.78	0.7	45.16	0.29	− 18.71
Jentjens, et al. (2005a) [67]	0	0	Water			1.55 ± 0.12	0.97 ± 0.05							
Jentjens, et al. (2004a) [21]	1.8	108	GLU	1.06 ± 0.08	0.93 ± 0.07	0.98 ± 0.1	0.67 ± 0.07	51.7	− 0.21	− 31.34	0.48	33.57	0.45	− 31.47
Jentjens, et al. (2004a) [21]	1.8	108	GLU:SUC 2:1	1.25 ± 0.07	1.12 ± 0.06	1.09 ± 0.12	0.58 ± 0.03	62.2	− 0.3	− 51.72	0.78	54.55	0.34	− 23.78
Jentjens, et al. (2004a) [21]	1.8	108	GLU:MALTOSE 2:1	1.06 ± 0.08	0.94 ± 0.05	1.07 ± 0.1	0.66 ± 0.03	52.2	− 0.22	− 33.33	0.58	40.56	0.36	− 25.17
Jentjens, et al. (2004a) [21]	0	0	Water			1.43 ± 0.15	0.88 ± 0.05							
Jentjens, et al. (2005b) [66]	1.2	72	GLU	0.77 ± 0.04	0.7 ± 0.03	1.53 ± 0.08	0.58 ± 0.05	58.3	− 0.19	− 32.76	0.54	31.95	0.16	− 9.47
Jentjens, et al. (2005b) [66]	1.2	72	SUC	0.98 ± 0.04	0.94 ± 0.04	1.31 ± 0.11	0.62 ± 0.06	78.3	− 0.15	− 24.19	0.56	33.14	0.38	− 22.49
Jentjens, et al. (2005b) [66]	1.2	72	GLU:SUC 1:1	0.9 ± 0.07	0.85 ± 0.03	1.43 ± 0.12	0.61 ± 0.05	70.8	− 0.16	− 26.23	0.59	34.91	0.26	− 15.38
Jentjens, et al. (2005b) [66]	2.4	144	GLU:SUC 1:1	1.2 ± 0.07	1.11 ± 0.03	1.33 ± 0.07	0.52 ± 0.04	46.3	− 0.25	− 48.08	0.75	44.38	0.36	− 21.3
Jentjens, et al. (2005b) [66]	0	0	Water			1.69 ± 0.1	0.77 ± 0.06							
Hearris, et al. (2022) [25]	2	120	MALTO:FRUC 1:0.8	1.56 ± 0.16	1.45 ± 0.07			72						
Hearris, et al. (2022) [25]	2	120	MALTO:FRUC 1:0.8	1.58 ± 0.13	1.44 ± 0.16			72						
Hearris, et al. (2022) [25]	2	120	GLU:FRUC 1:0.8	1.59 ± 0.08	1.5 ± 0.1			75						
Hearris, et al. (2022) [25]	2	120	MALTO:FRUC 1:0.8	1.66 ± 0.02	1.52 ± 0.13			75						
Pfeiffer, et al. (2010a) [89]	1.8	108	MALTO:FRUC 2:1	1.44 ± 0.29	1.28 ± 0.26	0.82 ± 0.32	0.53 ± 0.16	71.1	− 0.29	− 54.72	0.75	55.56	0.53	− 39.26
Pfeiffer, et al. (2010a) [89]	1.8	108	MALTO:FRUC 2:1	1.42 ± 0.23	1.24 ± 0.23	0.72 ± 0.21	0.59 ± 0.16	68.9	− 0.23	− 38.98	0.61	45.19	0.63	− 46.67
Pfeiffer, et al. (2010a) [89]	0	0	Water			1.35 ± 0.17	0.82 ± 0.32							
Pfeiffer, et al. (2010b) [65]	1.55	93	GLU:FRUC 2:1	1.25 ± 0.15	1.03 ± 0.11	1.23 ± 0.29	0.58 ± 0.21	66.5	− 0.31	− 53.45	0.62	37.8	0.41	− 25
Pfeiffer, et al. (2010b) [65]	1.55	93	GLU:FRUC 2:1	1.34 ± 0.27	1.14 ± 0.16	1.12 ± 0.34	0.57 ± 0.22	73.5	− 0.32	− 56.14	0.62	37.8	0.52	− 31.71
Pfeiffer, et al. (2010b) [65]	0	0	Water			1.64 ± 0.58	0.89 ± 0.29							
Jeukendrup, et al. (1997) [84]	1.06	63.6	GLU	0.95 ± 0.07	0.83 ± 0.03	1.15 ± 0.05	0.62 ± 0.04	77.8						
Jeukendrup, et al. (2006) [64]	1.5	90	GLU	1.24 ± 0.04	1.32 ± 0.04			88						
Jeukendrup, et al. (2006) [64]	1.5	90	GLU:FRUC 2:1	1.4 ± 0.08	1.49 ± 0.06			99.3						
Rowlands, et al. (2012) [76]	0.8	48	GLU		0.55 ± 0.13	1.12 ± 0.72	0.67 ± 0.25	68.8	− 0.1	− 14.93	0.17	11.33	0.38	− 25.33
Rowlands, et al. (2012) [76]	0	0	Water			1.5 ± 0.62	0.77 ± 0.28							

Study	Avg ingestion rate	CHO (g·h^−1^)	CHO solution	Peak Exo CHO Ox (g·min^−1^)	Avg Exo CHO Ox (g·min^−1^)	Avg Endo CHO Ox (g·min^−1^)	Fat Ox Rate (g·min^−1^)	Ox efficiency (%)	Δ Fat Ox (g·min^−1^)	Δ Fat Ox (%)	↑ Total CHO Ox (g·min^−1^)	↑ Total CHO Ox (%)	Δ Endo CHO Ox (g·min^−1^)	Δ Endo CHO Ox (%)
	Amount	Amount	(Ratio)	Mean (± SD)	Mean (± SD)	Mean (± SD)	Mean (± SD)	(Mean)	(Mean)	(Mean)	(Mean)	(Mean)	(Mean)	(Mean)
Rowlands, et al. (2011) [63]	0.8	48	GLU		0.54 ± 0.20	1.04 ± 0.70	0.72 ± 0.25	67.5	− 0.07	− 9.72	0.11	7.48	0.43	− 29.25
Rowlands, et al. (2011) [63]	0	0	Water			1.47 ± 0.85	0.79 ± 0.23							
Odell, et al. (2022) [88]	0.8	48	GLU		0.55 ± 0.03	1.29 ± 0.25	0.56 ± 0.08	68.8						
Odell, et al. (2020) [87]	0.8	48	LACTOSE	0.65 ± 0.19	0.56 ± 0.19			70						
Odell, et al. (2020) [87]	0.8	48	SUC	0.71 ± 0.13	0.61 ± 0.1			76.3						
Venables, et al. (2008) [62]	1.1	66	MALTOSE	1.01 ± 0.24	0.89 ± 0.23	1.2 ± 0.25	0.68 ± 0.19	80.9	− 0.23	− 33.82	0.47	29.01	0.42	− 25.93
Venables, et al. (2008) [62]	1.1	66	TREHALOSE	0.73 ± 0.22	0.62 ± 0.17	1.29 ± 0.33	0.79 ± 0.19	56.4	− 0.12	− 15.19	0.29	17.9	0.33	− 20.37
Venables, et al. (2008) [62]	0	0	Water			1.62 ± 0.28	0.91 ± 0.19							
Barber, et al. (2020) [61]	1.5	90	MALTO:GLU 1:1.38	0.9 ± 0.5	0.71 ± 0.26	2.26 ± 0.38	0.43 ± 0.09	47.4						
Barber, et al. (2020) [61]	1.5	90	MALTO:FRUC 1:1.38	1.1 ± 0.3	0.87 ± 0.28	2.28 ± 0.43	0.31 ± 0.15	57.7						
Rowlands, et al. (2008) [77]	0.6	36	MALTO		0.49 ± 0.09	1.49 ± 0.7	0.55 ± 0.23	81.7						
Rowlands, et al. (2008) [77]	0.9	54	FRUC:MALTO 0.5:1		0.73 ± 0.19	1.48 ± 0.71	0.51 ± 0.21	81.1						
Rowlands, et al. (2008) [77]	1.1	66	FRUC:MALTO 0.8:1		0.84 ± 0.22	1.49 ± 0.77	0.44 ± 0.22	76.4						
Rowlands, et al. (2008) [77]	1.3	78	FRUC:MALTO 1.2:1		0.78 ± 0.31	1.41 ± 0.78	0.46 ± 0.22	60						
Yeo, et al. (2005) [78]	0.8	48	GLU		0.57 ± 0.04	1.27 ± 0.13	0.93 ± 0.11	71.3	− 0.2	− 21.51	0.72	64.29	− 0.15	13.39
Yeo, et al. (2005) [78]	0	0	Water			1.12 ± 0.04	1.13 ± 0.17							
Wallis, et al. (2005) [79]	1.8	108	MALTO	1.5 ± 0.07	0.96 ± 0.07	1.38 ± 0.12	0.7 ± 0.06	53.3	− 0.18	− 25.71	0.4	20.62	0.56	− 28.87
Wallis, et al. (2005) [79]	1.8	108	MALTO:FRUC 2:1	1.06 ± 0.08	1.38 ± 0.06	1.23 ± 0.11	0.6 ± 0.04	76.7	− 0.28	− 46.67	0.67	34.54	0.71	− 36.6
Wallis, et al. (2005) [79]	0	0	Water			1.94 ± 0.11	0.88 ± 0.06							
Wallis, et al. (2006) [91]	1.5	90	GLU	0.7 ± 0.08	0.8 ± 0.1	1.87 ± 0.18	0.5 ± 0.07	53.3	− 0.15	− 30	0.42	18.67	0.38	− 16.89
Wallis, et al. (2006) [91]	1.5	90	GLU	0.65 ± 0.06	1.02 ± 0.1	1.55 ± 0.17	0.62 ± 0.03	68	− 0.18	− 29.03	0.69	36.46	0.33	− 17.7
Wallis, et al. (2006) [91]	0	0	Water			2.25 ± 0.25	0.65 ± 0.07							
Wallis, et al. (2006) [91]	0	0	Water			1.88 ± 0.18	0.8 ± 0.05							
Podlogar, et al. (2022) [6]	2	120	MALTO:FRUC 1:0.8	1.6 ± 0.25	1.51 ± 0.22	2.15 ± 0.3		75.5						
Podlogar, et al. (2022) [6]	1.5	90	MALTO:FRUC 2:1	1.36 ± 0.17	1.29 ± 0.16	2.2 ± 0.33		86						
Podlogar, et al (2025) [59]	1.5	90	GLU:FRUC 2:1	0.90 ± 0.15	0.87 ± 0.14	0.88 ± 0.29	0.58 ± 0.02	58	− 0.04	− 6.90	0	0	0.03	3.53
Podlogar, et al (2025) [59]	1.09	65.4	GLU:FRUC 2:1	0.91 ± 0.19	0.90 ± 0.18	0.85 ± 0.31	0.62 ± 0.31	82.6						
Jentjens, et al. (2004b) [83]	2.4	144	GLU	1.18 ± 0.04	1.02 ± 0.04	1.05 ± 0.06	0.67 ± 0.05	42.5	− 0.19	− 28.36	0.6	40.82	0.42	− 28.57
Jentjens, et al. (2004b) [83]	2.4	144	GLU:FRUC:SUC 2:1:1	1.7 ± 0.07	1.54 ± 0.06	0.76 ± 0.12	0.59 ± 0.08	64.2	− 0.27	− 45.76	0.83	56.46	0.71	− 48.3
Jentjens, et al. (2004b) [83]	0	0	Water			1.47 ± 0.06	0.86 ± 0.05							
Ravikanti et al. (2025) [2]	1	60	MALTO	1.0 ± 0.1	0.89 ± 0.11		1.07 ± 0.22	89						
Ravikanti et al. (2025) [2]	1.5	90	MALTO:FRUC 2:1	1.41 ± 0.12	1.31 ± 0.18		0.90 ± 0.27	87	− 0.17	− 15.9	0.37	17.7		
Ravikanti et al. (2025) [2]	2	120	MALTO:FRUC 1:1	1.77 ± 0.13	1.68 ± 0.16		0.67 ± 0.20	84	− 0.40	− 37.4	0.98	46.9		
Achten, et al. (2007) [80]	1.1	66	SUC	0.92 ± 0.03	0.87 ± 0.03	1.19 ± 0.11		79.1			0.54	35.53	0.33	− 21.71
Achten, et al. (2007) [80]	0	0	Water			1.52 ± 0.08								

Ratios presented in the ‘CHO solution’ column indicate the relative proportion of carbohydrate sources provided

Values are presented as mean (± SD) where available. Blank cells indicate that the variable was not measured or not reported in the original source. Shaded rows represent placebo (0 g·min^–1^; 0 g·h^–1^ CHO) control conditions

Multiple rows from the same study reflect different carbohydrate formulations or experimental conditions within that study

CHO, carbohydrate; Exo, exogenous; Endo, endogenous; Ox, oxidation; Avg, average; Δ, change or difference; SD, standard deviation. GLU, glucose; FRUC, fructose; MALTO, maltodextrin; SUC, sucrose; DEX, dextrose; TRE, trehalose; LACT, lactose; Peak Exo CHO Ox, peak exogenous carbohydrate oxidation; Avg Exo CHO Ox, average exogenous carbohydrate oxidation; Avg Endo CHO Ox, average endogenous carbohydrate oxidation; Fat Ox rate, fat oxidation rate; Ox efficiency, oxidation efficiency; ↑ Total CHO Ox, increase in total carbohydrate oxidation

**Fig. 1:**
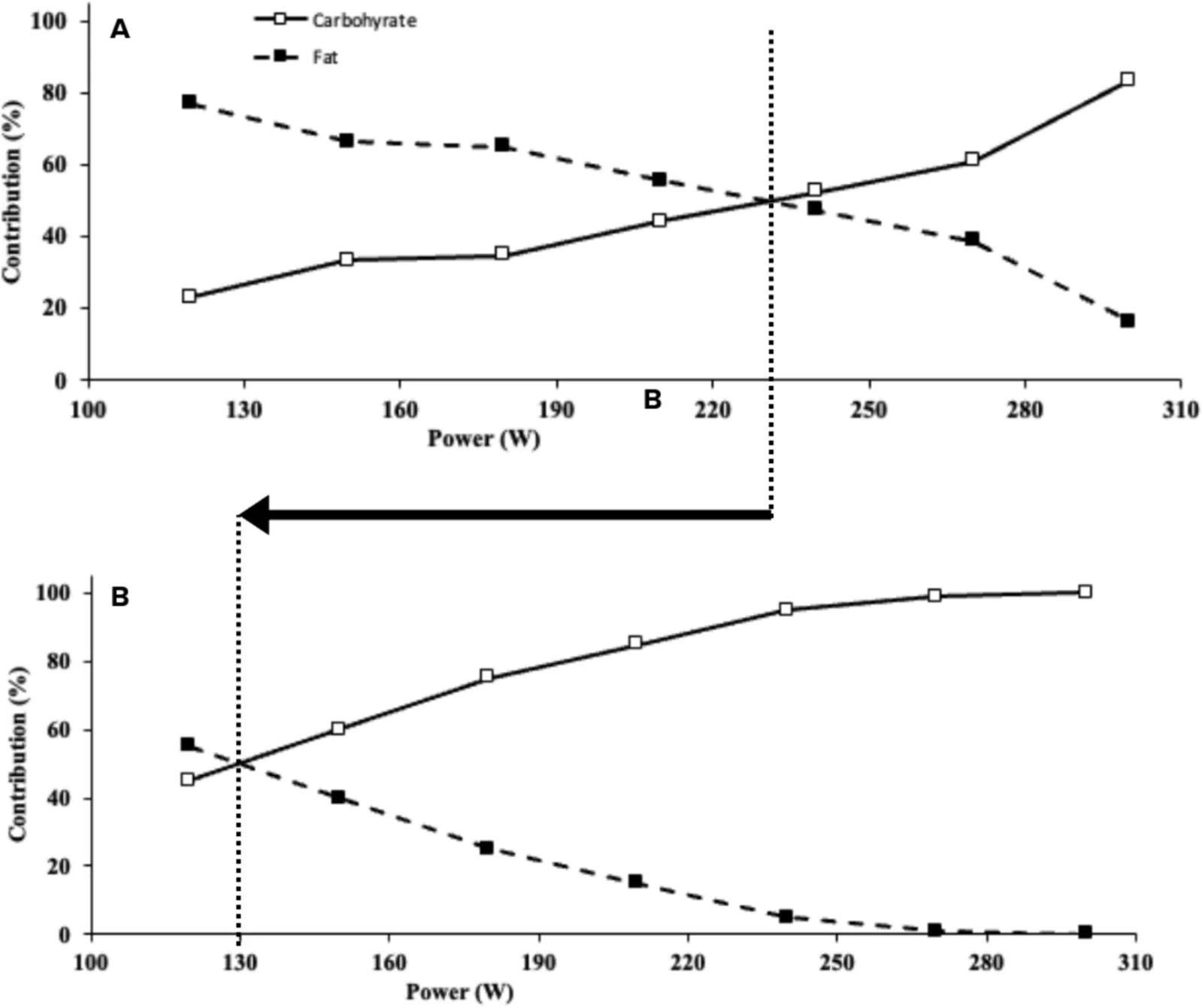
Theoretical illustration of how ultra-high carbohydrate ingestion may shift the crossover point (vertical dotted line) to the left. Historically, when overall substrate availability limited endurance performance, shifting the curve to the right was considered favourable (**A**). With modern fuelling strategies effectively removing substrate constraints, potential gains may now come from subtle improvements in oxidative efficiency and metabolic economy (**B**)

These data may provide a plausible explanation for the anecdotal success of U-HC ingestion rates. High exogenous carbohydrate intake shifts oxidation towards carbohydrates, lowering oxygen cost. This perspective challenges the long-standing paradigm in endurance sports, which has emphasised benefits of delaying carbohydrate depletion by promoting fat oxidation, shifting the so-called crossover point to the right [48] (Fig. 1). Conversely, U-HC ingestion rates may mean intentionally shifting the crossover point to the left, favouring carbohydrate oxidation, even at lower intensities.

**Fig. 2:**
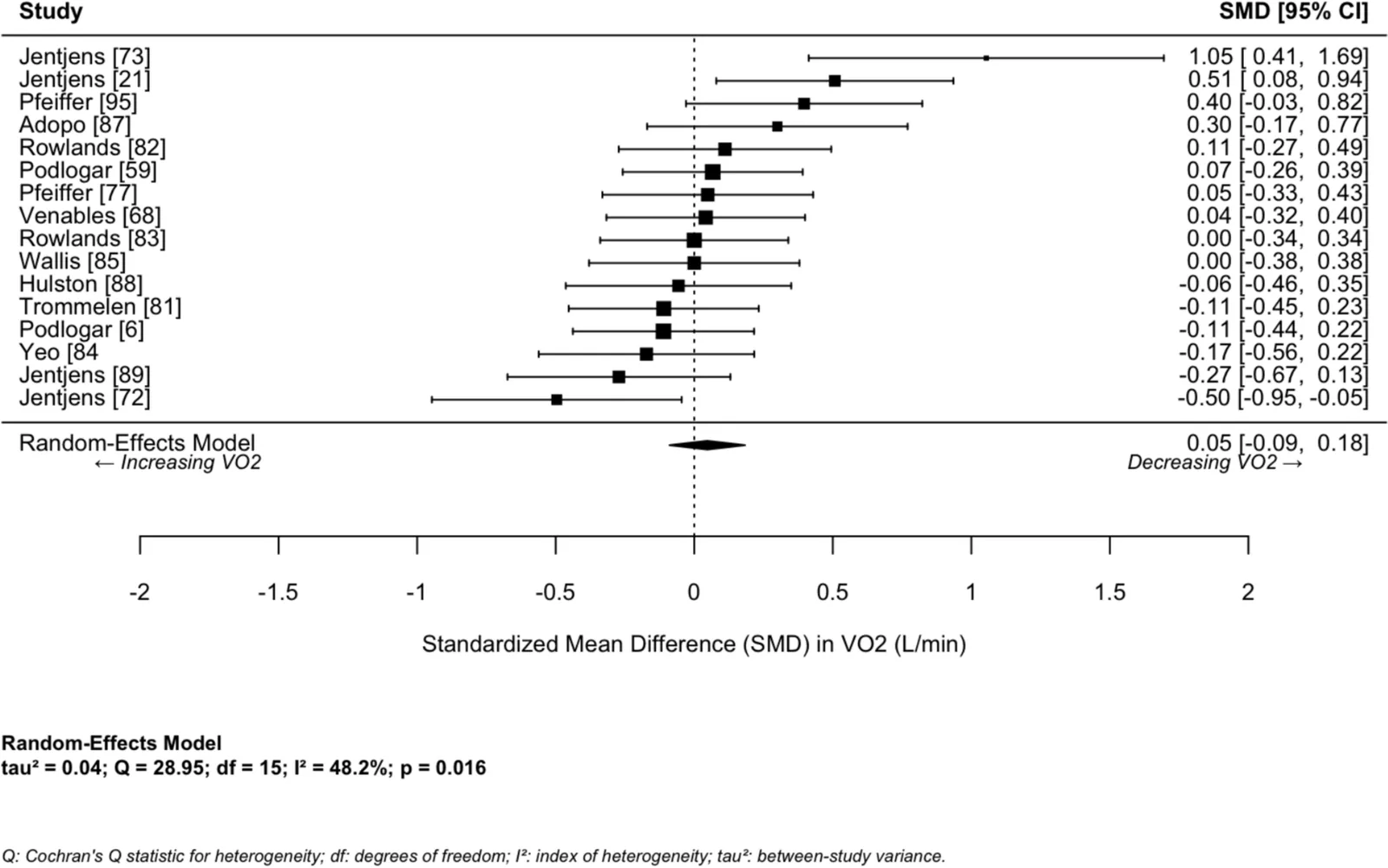
Standardised mean differences (SMD) in V̇O_2_ (L·min⁻^1^) from the lowest to the highest carbohydrate intakes in studies reporting the mean and standard deviation of VO_2_ during exercise (*n* = 16). Each line represents an individual study with its 95% confidence interval. Negative values indicate a higher oxygen cost (worse exercise economy), whereas positive values indicate a lower oxygen cost (better exercise economy). The diamond represents the overall random effects estimate from the multilevel model (SMD = 0.05 [95% CI: − 0.09 to 0.18]), providing no clear evidence of a consistent effect of carbohydrate intake on V̇O_2_. Between-study heterogeneity was moderate (*τ*^2^ = 0.04; *I*^2^ = 48.2%; *Q* = 28.95, df = 15, *p* = 0.016), indicating meaningful variability in true effects across studies. SMDs were derived from studies included in this review (Table 1) in which mean V̇O_2_ and corresponding standard deviation data were reported. Where such data were available, the SMD was calculated by comparing V̇O₂ at either a zero-carbohydrate condition or the lowest reported ingestion rate against the highest carbohydrate ingestion rate, whichever was applicable, each with their respective standard deviations. This approach was adopted to extract the most meaningful contrast between conditions

This shift towards greater carbohydrate reliance may prioritise glycolytic and lactate-derived substrates, meaning athletes reduce fractional utilization of V̇O_2_max, improve economy and delay fatigue. Recent data from Ravikanti et al. [2] support this, as they showed a 3.6% improvement in running economy when consuming 120 g·h⁻^1^ versus 60 g·h⁻^1^ in elite marathon runners. Figure 2 displays the standardised mean differences (SMD) in oxygen consumption (VO_2_, L·min⁻^1^) comparing the highest and lowest carbohydrate intakes across 16 studies. The overall pooled effect was trivial and non-significant (SMD = 0.05 [95% CI: − 0.09 to 0.18]), with no clear evidence of a consistent effect of carbohydrate intake on VO_2_. Between-study heterogeneity was moderate (*τ*^2^ = 0.04;* I*^2^ = 48.2%; *Q* = 28.95, df = 15, *p* = 0.016), reflecting meaningful variability in true effects across studies. However, most studies examined intakes well below the ultra-high carbohydrate range, limiting conclusions at these doses. These data suggest that carbohydrate intake is unlikely to substantially influence exercise economy across the intake ranges currently represented in the literature, though the effect of ultra-high carbohydrate intakes on oxygen cost and exercise efficiency warrants further investigation.

However, it is important to note that improved oxygen efficiency or shifts in substrate utilization do not necessarily translate into meaningful performance benefits. Apparent improvements in economy based on absolute V̇O₂ may reflect substrate-dependent differences in oxygen cost rather than true enhancements in mechanical or physiological efficiency [49]. Furthermore, the proposal that U-HC intake may intentionally shift the crossover point to the left remains speculative. The crossover point is highly variable and strongly influenced by habitual diet and training state, meaning such shifts are often not unique to acute high carbohydrate availability [11]. Whether this translates into a performance advantage, in lesser-trained athletes (e.g. V̇O_2_max < 60 mL·kg·min⁻^1^) where absolute V̇O₂ is much lower, also remains to be established.

### Carbohydrate-to-Lactate Interplay in Trained Metabolism?

A possible second mechanism is via lactate production. High rates of fructose ingestion within glucose–fructose mixtures increase systemic lactate availability [50]. Fructose is rapidly converted to glucose and lactate, increasing the circulating lactate pool available for oxidation. Lactate, once viewed merely as a metabolic by-product, is now well-recognised as a central oxidative fuel [51]. Through the lactate shuttle, lactate generated in glycolytic tissues can be transported to and oxidised by highly aerobic cells such as type I muscle fibres and cardiac tissue. It is worth noting, however, that this mechanism is contingent on sufficient fructose absorption. Intestinal fructose absorption via GLUT5 is generally considered to saturate at approximately 30–60 g·h⁻^1^ [14, 21, 52], and at U-HC ingestion rates employing a 1:0.8 or 1:1 glucose:fructose ratio, fructose intake approaches this upper boundary. Fructose ingested beyond the maximum absorption rate would not enter systemic circulation and therefore could not contribute meaningfully to hepatic lactate production or systemic lactate availability. As such, the lactate shuttle mechanism may be most relevant at ingestion rates where fructose absorption remains intact, and its contribution at true U-HC rates remains uncertain. Notably, lactate shuttling may be upregulated in elite endurance athletes, who have elevated monocarboxylate transporter 1 and 4 expression, and greater mitochondrial volume, than non-athletes or those with lower training status [53]. These adaptations may enable more efficient lactate uptake and conversion to pyruvate, thereby fuelling oxidative phosphorylation. Plausibly, fructose-derived hepatic lactate production and an elevated capacity for lactate shuttling in elite athletes may result in heightened exogenous carbohydrate oxidation rates with U-HC ingestion not seen in lesser-trained populations, though this remains speculative and warrants direct investigation.

### A Possible Brain-Derived Effect?

A third mechanism through which U-HC ingestion rates might enhance endurance performance is via the brain. Evidence from carbohydrate mouth rinse studies suggests the mere presence of carbohydrate in the oral cavity can improve performance, suggesting a brain-derived mechanism independent of peripheral substrate availability [54]. This effect is thought to be mediated via oral carbohydrate receptors, specifically the T1R2/T1R3 sweet taste receptor complex, which triggers afferent signalling to reward and motor control centres in the brain independent of metabolic carbohydrate status [55]. It is acknowledged that mouth rinse benefits are most consistently observed under carbohydrate-depleted or fasted conditions, and that most of this evidence derives from shorter-duration or high-intensity protocols where metabolic limitation is unlikely to be a primary constraint. In contrast, during longer-duration endurance exercise, the ergogenic effects of carbohydrate ingestion appear to be driven primarily by the maintenance of blood glucose, with any central effects likely secondary [11]. Nevertheless, we propose that continuous high carbohydrate availability during U-HC feeding may sustain oral and gut carbohydrate receptor stimulation throughout prolonged exercise, potentially contributing to maintained ratings of perceived exertion (RPE) attenuation across the full duration of an event. In support, ratings of perceived exertion (RPE) are consistently reduced with carbohydrate ingestion. For example, Learsi et al. [56] reported significantly lower RPE during 105 min of submaximal constant load exercise with carbohydrate ingestion, which resulted in significantly faster subsequent 10-km TT performance. Similarly, Steiner et al. [57] observed a non-significant tendency towards reduced RPE during incremental cycling (*p* = 0.07) and a modest 2.1% increase in work output at a fixed RPE during a subsequent constant-load trial with carbohydrate supplementation.

Together, these findings indicate that carbohydrate availability may modulate central fatigue and perceptual responses, potentially influencing pacing or motivation. Such effects may be more pronounced during shorter or higher-intensity efforts; during prolonged endurance events such as Ironman triathlons or Grand Tour cycling races, metabolic factors such as blood glucose maintenance are likely the primary drivers of any ergogenic benefit. Whether U-HC ingestion results in a proportionally greater central effect remains unknown. It is plausible that ‘ultra-high’ availability could further attenuate central fatigue or lower RPE; however, a dose–response relationship is yet to be established.

## Conclusions

The three models discussed present plausible, yet still speculative, explanations for the purported benefits of U-HC intake. While each offers a mechanistic rationale, these hypotheses remain untested in real-world settings. However, if correct, the concepts are likely not mutually exclusive and may operate synergistically to contribute to an overall performance advantage. To advance our understanding, we must look beyond traditional paradigms such as glycogen sparing, hypoglycaemia prevention and maximising exogenous oxidation rates. Despite its rising popularity among elite endurance athletes, the U-HC fuelling trend remains supported largely by anecdotal reports rather than empirical evidence. Indeed, most research suggests that the performance benefit of carbohydrate ingestion shows diminishing returns beyond 90 g·h⁻^1^, with no clear dose–response effect at higher ingestion rates in most individuals [32].

Importantly, there are clear inter-individual differences in exogenous carbohydrate oxidation capacity, and it stands to reason that some elite athletes possess the physiological ability to oxidise carbohydrates at exceptionally high rates. Hearris et al. [25] reported a range of oxidation rates between ~ 1.3 g·min⁻^1^ and 1.9 g·min⁻^1^ across participants despite standardized ingestion protocols at 120 g·h⁻^1^. To contextualise this variability, an athlete oxidising at the upper end of this range (~ 1.9 g·min⁻^1^) would be utilising approximately 114 g·h⁻^1^, approaching near-complete utilization of a 120 g·h⁻^1^ ingestion rate. In contrast, an athlete at the lower end (~ 1.3 g·min⁻^1^) would oxidise only ~ 78 g·h⁻^1^, leaving a substantial proportion of ingested carbohydrate unoxidised and therefore unlikely to confer additional metabolic benefit. This suggests that U-HC strategies may only be fully utilised by small few at the uppermost end of the oxidation distribution, and that for most athletes, ingestion rates of 120 g·h⁻^1^ are likely to exceed their oxidative capacity. More recently, Ijaz et al. [58] highlighted that larger athletes exhibit greater absolute oxidation rates than their smaller counterparts, emphasising the role of individual physiology in substrate utilization.

Ideally, carbohydrate fuelling would be tailored to each athlete’s individual oxidation capacity. Recently, Podlogar et al. [59] demonstrated that by adjusting intake on the basis of individual glucose oxidation rates, athletes were able to achieve similar carbohydrate utilization while consuming 28% less and simultaneously reducing both perceived exertion and gastrointestinal fullness and discomfort. Nevertheless, assessing individual oxidation rates requires laboratory resources far beyond the reach of most athletes and coaches, and may be limited to exercise at stable intensity, rather than the quickly shifting metabolic demands found in competition events. Therefore, population-based guidelines must rely on average values, those most likely to (1) reflect typical oxidation capacity, (2) optimise performance outcomes and (3) minimise gastrointestinal distress across a wide range of athletes.

In addition to this, we must not overlook the potential long-term health implications of adopting a U-HC fuelling strategy, particularly when athletes chronically elevate overall carbohydrate consumption to train the gut. While the acute metabolic response to U-HC ingestion during exercise is distinct from chronic dietary carbohydrate intake, the two are difficult to disentangle in practice, as gut training protocols necessarily involve habitual high carbohydrate availability. In this regard, Prins et al. [60], reported that even moderately elevated chronic carbohydrate intake resulted in pre-diabetic fasting glucose patterns (> 100 mg/dL) in approximately 30% of athletes over 31 days, an effect that was reversible upon carbohydrate reduction. While this study examined chronic dietary intake rather than acute exercise fuelling per se, it raises important questions about the metabolic consequences of the sustained elevation in carbohydrate consumption that is likely to accompany any gut training programme, particularly among amateur athletes who may over-fuel before, during and after training sessions without the physiological demand to justify such intake.

Finally, no studies to date have examined the effects of U-HC intake across the full duration of events such as Ironman triathlons or ultramarathons. U-HC fuelling strategies do, however, hold promise in the context of recovery. High carbohydrate availability during exercise may play a key role in facilitating better day-to-day recovery, especially during periods of intensified training or in multistage races. These benefits are more likely to manifest when rapid turnaround between sessions or races is required. In this context, aggressive carbohydrate fuelling may contribute to improved training quality, reduced residual fatigue and better overall adaptation. However, this remains a distinct physiological goal from acute race-day performance, and further research is needed to determine the optimal application of U-HC strategies in such recovery-focused scenarios. In conclusion, while U-HC fuelling may hold potential in niche scenarios or for uniquely gifted athletes, we are far from having enough evidence to recommend this strategy broadly. Guidelines must be anchored in established science, with room for individualised optimization built on top. At present, this means prioritising evidence-based carbohydrate targets rather than pursuing untested trends. When reviewing the data presented here (Table 1), it becomes clear that increasing carbohydrate ingestion rates are associated with progressively lower oxidation efficiency. The glucose:fructose ratio plays the most important role in enhancing total exogenous carbohydrate oxidation. Examining the average oxidation values in Table 1 of all studies, ratios of 1:0.8 to 1:1 consistently yield the highest average oxidation rates (~ 88 ± 9 g·h⁻^1^), whereas more traditional 2:1 blends often produce lower averages (~ 65 ± 7 g·h⁻^1^). For most athletes, these observed averages offer a practical and evidence-based starting point for developing race-day nutrition strategies. A key consideration for practice is the growing tendency for amateur and recreational athletes to adopt U-HC intakes as a gold standard of performance nutrition. Given the absence of robust evidence supporting additional benefit beyond ~ 60–90 g·h⁻^1^ in this population, recommending U-HC strategies is premature and should not be actively encouraged until the evidence base matures. Future research should explore whether approaches such as 120 g·h⁻^1^ at a 1:0.8–1 ratio confer genuine performance benefit or whether they reflect the unique physiology of a metabolically gifted minority.

## Supplementary Information

Below is the link to the electronic supplementary material.

Supplementary file1 (DOCX 3044 KB)

## Declarations

### Availability of Data and Materials

No new data were generated for this manuscript. All data shown in Figs. 1 and 2 were extracted from previously published studies, the full details of which are provided in the manuscript references. All other content represents a narrative synthesis of existing literature, and no primary data were collected or analysed.
