# Supplementary material for: Fuelled or Fooled? Examining the Evidence and Mechanisms Behind Ultra-High Carbohydrate Intake in Endurance Athletes
Source: Sports Med. 2026 Jun 8;56(10):2367–88. doi: 10.1007/s40279-026-02462-z (PMC13615066; doi:10.1007/s40279-026-02462-z)
Supplement: Supplementary file 1 [file 40279_2026_2462_MOESM1_ESM.docx]

**Study Inclusion Criteria**

Online databases, including PubMed, Web of Science, and Scopus, were searched for relevant studies. Studies were included if they were conducted on human participants, available in English, and published in peer-reviewed journals. The inclusion criteria also required studies to involve continuous exercise lasting more than 60 minutes in normothermic conditions (15–25 °C) with a carbohydrate supplementation intervention and to report exogenous carbohydrate oxidation rates. Studies were also required to report one or more of the specified outcome measures, including average ingestion rates; oxidation rates; % of VO2max; absolute VO2; absolute Watts; W/kg; CHO type; CHO solution; fluid concentration/osmolality. Studies were excluded if they were: conducted on animals, or not involving human participants; not related to carbohydrate supplementation and exercise performance; involving ingestion of fats or protein; involving exercise duration less than 60 minutes; lacking necessary outcome measure data; not published in peer-reviewed journals; reporting insufficient methodological details.

The following search terms were entered into the relevant databases specified: ‘carbohydrate AND endurance AND exercise;’ ‘carbohydrate ingestion rate AND endurance performance;’ ‘exogenous carbohydrate oxidation rates AND prolonged exercise;’ ‘endogenous carbohydrate oxidation rates AND endurance sport;’ ‘fat oxidation rates AND prolonged exercise;’ ‘carbohydrate solution AND exercise.’

The specified search terms were combined to capture studies that met multiple criteria. Titles and abstracts were screened against the eligibility criteria. Studies that met the criteria or could not yet be eliminated were retrieved to assess the full text. After reviewing the retrieved full-text studies, relevant data were then extracted for analysis from studies that met the criteria.

All statistical analyses and forest plot visualizations were conducted using RStudio (version 2026.01.2+418) utilizing the metafor package.
